# QTc Prolongation Risk Evaluation in Female COVID-19 Patients Undergoing Chloroquine and Hydroxychloroquine With/Without Azithromycin Treatment

**DOI:** 10.3389/fcvm.2020.00152

**Published:** 2020-09-02

**Authors:** Sarah Grewal, Lior Jankelson, Marcel P. H. van den Broek, Martin Cour, Gloria Bachmann, John B. Kostis, Kamana Misra

**Affiliations:** ^1^Pharmacovigilance, ContraRx, NJ, United States Edison, NJ, United States; ^2^NYU Langone Health, New York University School of Medicine, New York, NY, United States; ^3^Department of Clinical Pharmacy, St. Antonius Hospital, Utrecht, Netherlands; ^4^Hospices Civils de Lyon, Hôpital Edouard Herriot, Service de Médecine—Intensive Réanimation, Lyon, France; ^5^Women's Health Institute, Rutgers Robert Wood Johnson Medical School, New Brunswick, NJ, United States; ^6^Cardiovascular Institute, Rutgers Robert Wood Johnson Medical School, New Brunswick, NJ, United States

**Keywords:** COVID, QTc changes, hydroxychloroquine, chloroquin, azithromycin (AZM), QTc, hydroxychloroquine (HCQ), women

## Abstract

Women have higher risk for developing TdP in response to ventricular repolarization prolonging drugs. Hundreds of trials are administering chloroquine and hydroxychloroquine with/without azithromycin to COVID-19 patients. While an overall prolonged QTc has been reported in COVID-19 patients undergoing these treatments, the question on even higher QTc elevation risk in thousands of female COVID-19 patients undergoing these treatments remains unanswered. We therefore explore data reported and shared with us to evaluate safety and efficacy of antimalaria pharmacotherapies in female COVID-19 patients. Although we observed longer mean QTc intervals in female patients in 2 of the 3 cohorts reviewed, the sex disproportionality in COVID-19 hospitalizations precludes a clear sex mediated QTc interval elevation risk association in the female COVID-19 patients undergoing acute treatment regimens. Adoption of study designs that include observation of sex mediated differential triggering of cardiac electrical activity by these drugs is warranted.

## Introduction

Female gender is a known risk factor for QTc prolongation and one of the highest pro-arrhythmic risk factors (1–3). Women are at a significantly greater risk than men for developing the potentially fatal ventricular arrhythmia torsades de pointes (TdP) in response to certain drugs that prolong ventricular repolarization (1, 4). TdP occurs three times more commonly in women than in men, and female gender is also an independent risk factor for the incidence of syncope and sudden death in the inherited long QT syndrome (LQTS) (5, 6).

In the current coronavirus disease 2019 (COVID-19) pandemic crisis, without validated treatment options available for management, hundreds of trials are administering chloroquine and hydroxychloroquine with/without azithromycin to COVID-19 patients in monitored settings, while associated benefits and risks remained debated. Chloroquine, hydroxychloroquine, and azithromycin are individually implicated in prolonging corrected heart rate (QTc), a predictor of TdP (7–10). Concurrent use of QTc altering drugs can result in synergistic increase in risk of ventricular arrhythmias and sudden death (11, 12), a setting recreated by the current COVID-19 treatment regimens.

Factors associated with increased QT prolongation cardiotoxicity risk in females include hormonal mediated differentiation in cardiac electrical activities, greater genetic predisposition for Long QT Syndrome (LQTS) and a higher propensity for drug acquired LQTS driven by drug-drug Interactions (13). The baseline QTc is longer in women than in men (14–16). Endogenous estrogen is associated with QTc lengthening, while testosterone and progesterone shorten the action potential (16, 17). Small QTc prolongation also has been reported with some fourth generation oral contraceptives (18). Women have a higher predisposition to genetic mutations that potentiate TdP and have a higher risk of TdP with Long QT syndrome (LQTS) type 1 and type 2, caused by mutations in potassium channel gene KCNQ1 (KvLQT1) and mutations in potassium channel gene KCNH2 (also known as hERG), respectively (19, 20).

Furthermore, female gender is increasingly recognized as an independent risk factor for acquired LQTS which mainly occurs on exposure to an environmental stressor, most common being an adverse drug reaction leading to drug induced LQTS (DI-LQTS). The mechanisms underlying QT prolongation by medications in acquired LQTS almost always involve blockage of the inward potassium rectifier (IKr) channel, also known as the human ether-a-go-go-related gene (hERG) channel (21). IKr channel controls the movement of potassium out of the myocytes and conducts a rapid IKr current, a critical current in the phase 3 repolarization of the cardiac action potential (22). LQTS and the QT interval prolongation relative to the administration of IKr blockers is greater in women and accompanied by a propensity of drug-induced polymorphic ventricular arrhythmia (5, 23). The estrogen-mediated reduced repolarization reserve in women is believed to be responsible for their higher susceptibility to DI-LQTS (5).

Although the higher risk in females is well-recognized, mechanisms underlying these sex-based risk differences are notably poorly understood. Using a combined experimental and computational approach using “male” and “female” computational model representations of human ventricular cardiac myocytes, a recent study provides first evidence linking structure to function mechanisms underlying higher risk for acquired long-QT-dependent arrhythmias in females (24). Structural modeling presented two distinct, plausible mechanisms of estrogen action enhancing torsadogenic effects: estradiol interaction with hERG mutations in the pore loop containing G604 or with common TdP-related blockers in the intra-cavity binding site. The model predicted increased risk for arrhythmia in females when acute sympathetic nervous system discharge was applied in the settings of both inherited and acquired long-QT syndrome.

Another study models prediction of potential cardiac adverse events caused by combination COVID-19 treatments by combining simulations of pharmacokinetics (PK) with quantitative systems pharmacology (QSP) modeling of ventricular myocytes (25). Their simulation results predicted that drug combinations can lead to greater cellular action potential prolongation compared to drugs given in isolation. The simulations of different patient groups also predicted that females with pre-existing heart disease are especially susceptible to drug-induced arrhythmias, compared males with disease or healthy individuals of either sex.

Despite the high cardiotoxicity risk associated with combination treatments of Chloroquine, hydroxychloroquine and azithromycin in female COVID-19 patients, clinical data supportive of this risk outcome is still not available. We provide first look at clinical QTc data elongation patterns in male vs. female COVID-19 patients from multiple clinical trials across different geographical regions.

## Results and Discussion

With surveillance of the COVID-19 management interventions, studies are now reporting safety concerns, including the risk of QTc prolongation in patients receiving these treatments (Table 1). Re-iterating the predicate hypotheses, all these studies report clinically relevant QTc interval increase in COVID-19 patients receiving these pharmacotherapies and recommend continuous QTc interval monitoring and strict cutoffs for therapy cessation (9, 26–32). While an overall prolonged QTc is observed in COVID-19 patients undergoing these treatments, the question on even higher QTc elevation risk in thousands of female COVID-19 patients undergoing these treatments remains unanswered. We therefore look deeper into the data reported (28, 30) and additional data shared with us (9, 26, 27, 29, 31) to assess if sex mediated disparities in the QTc alterations exist in COVID-19 treatments.

**Table 1:** Corrected QT (QTc) Interval prolongation review from different studies.

**Drug/s**	**N**	**Sex F, (%)**	**Baseline QTc (ms)**	**QTc max (ms)**	**ΔQTc (ms)**	**QTc> 500 (%)**	**TdP**	**QTc based Rx Red./Term. (n)**	**Efficacy**	**Study (ref.)**
CQ	95	(34)	432 (360–505)	466 (383–549)	34 (25–43)	23	No	22	Unknown	22
Total	40	(20)	414 (392–428)	454 (420–480)	35 (10–66)	17.5	No	17	Unknown	27
HCQ	22									
HCQ + AZ	18									
HCQ + AZ	251	(25)	439 ± 29	473 ± 36	34 ± 35	13	1 VT (TdP?)	8	Unknown	9
Total	90	(49)	455 (430–474)	476 (445–500)	21 (1–39)	20	1TdP	10	Unknown	24
HCQ	90		474 (454–487)	479.5 (443.5–501.5)	5.5 (14–31)					
HCQ + AZ	53		442 (427–461)	458 (449–492)	23 (10–40)					
CQ + AZ + Os	81	(25)	424.7 (27.4)	Unknown	Unknown	15	2 VT	Unknown	Maybe	26
Low Dose	40		421.9 (24.0)	Unknown	Unknown	11			Lethality	
High Dose	41		427.8 (31.0)	Unknown	Unknown	18.9				

In recent report from on New York and Italy COVID-19 patient cohort, Chorin et al. (9, 29) reported significantly prolonged QTc intervals in COVID-19 patients treated with hydroxychloroquine and azithromycin. Additional data shared with us on a cohort of 251 patients shows that the average baseline QTc in male patients was 441 ± 30 ms while maximum QTc during treatment (QTc max) was 476 ± 36 ms (Table 2A). For the female patients, mean baseline QTc of 438 ± 26 and QTc max of 468 ± 38 were observed. This translates into an average increase of 35 ms in the male patients and 30 ms in female patients after treatment with hydroxychloroquine and azithromycin. Sample QTc elevation illustrations in two female patients are shown (Table 2D).

**Table 2:** (A–C) QTc prolongation comparison in male and female Coronavirus Disease 2019 patients.

	**Total**	**Males**	**Females**
**(A) Chorin et al**. **(****9****)**			
Number	251	188	63
Baseline QTc (ms)	439 ± 29	441 ± 30	438 ± 26
QTc max (ms)	473 ± 36	476 ± 36	468 ± 38
ΔQTc (ms)	34 ± 35	NA	NA
**(B) van den Broek et al**. **(****26****)**			
Number	95	32	63
Baseline QTc (ms)	432 (360–505)	429 (369–609)	438 (346–514)
QTc max (ms) (95% CI)	466 (383–549)	461 (385–557)	476 (415–600)
ΔQTc (ms) (95% CI)	34 (25–43)	32 (21–44)	38 (22–54)
**(C) Bessière et al**. **(****31****)**			
Number	40	32	8
Baseline QTc (ms)	414 (392–428)	413 ± 30	411 ± 26
QTc max (ms)	454 (420–480)	452 ± 45	463 ± 29
ΔQTc (ms)	35 (10–66)	38 ± 36	52 ± 31
**(D)**			
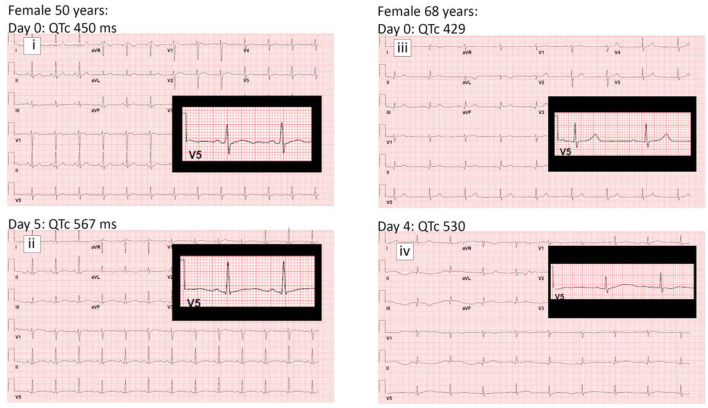			

*(D) QTc interval prolongation sample illustrations in 2 female patients. Inset images show magnification of lead V5. (i). Baseline ECG for a 50 years old female patient before the initiation of HY/AZ. QTc interval = 450 ms. (ii). QTc interval prolonged to 567 ms on day 5. (iii) Baseline ECG for another 68 years old female patient before the initiation of HY/AZ. QTc interval = 429 ms. (iv). QTc interval prolonged to 530 ms on day 4. NA, Not Available*.

Another retrospective, observational cohort conducted on 95 COVID-19 patients in Netherlands also reported clinically relevant prolonged QTc intervals in patients undergoing chloroquine therapy (26). Additional data shared with us reveals that female patients made up 34% of the cohort and their average baseline QTc of 438 ms (346–514) was elevated to 476 ms (415–600) post-chloroquine administration (manual interpretation). In male patients, a baseline mean QTc of 429 ms (369–609) increased to 461 ms (385–557) post-treatment. An increase of 32 ms in male patients (*n* = 63) and 38 ms in female patients (*n* = 32) was recorded during chloroquine treatment (Table 2B). Eight of 32 females (25%) had a QTC > 500 ms post-chloroquine vs. 14 of 64 males (22%).

Recent publication by Bessière et al. (31) on data from 49 COVID-19 ICU patients in France reveals QTc interval increase in 93% patients after the administration of the antiviral therapy with hydroxychloroquine alone or in combination with azithromycin. The overall median baseline QTc was 414 ms and max QTc after antiviral therapy was 454 ms. This cohort include 20% female patients and a 52 ms mean increase was observed in their baseline QTc average of 411 ± 26 ms to a maximal average value of 463 ± 29 during treatment. The baseline QTc average of 413 ± 30 in males was elevated to 452 ± 45 after treatment (Table 2C). None of the female patients showed QTc >500 ms.

Data from New York state department of health retrospective cohort of 1,438 patients hospitalized COVID-19 patients treatment with hydroxychloroquine, azithromycin, or both, indicated that cardiac arrest was more likely in patients receiving hydroxychloroquine and azithromycin, compared to hydroxychloroquine alone and azithromycin alone (27). Additional data (personal communication) on male and female patient distribution revealed QTc prolongation was observed in 13.0% male and 12.0% female patients in the HCQ and azithromycin cohort. In the HCQ alone cohort prolongation was observed in 16.8% males and 16.7% female patients. For the azithromycin alone cohort, they observed 7.8% percent prolongation in males and 9.2% prolongation in female patients and in the no treatment cohort, 11.7% prolongation in males and 5.1% prolongation in female patients.

## Conclusions

Although we observed longer mean QTc intervals in female patients in 2 of the 3 cohorts reviewed, no conclusive sex mediated QTc interval elongation is apparent amongst the COVID-19 patients undergoing acute chloroquine and hydroxychloroquine with/without azithromycin treatment regimens (9, 26, 27, 29, 31). Since a greater proportion of COVID-19 patients admitted to the hospitals are males, the sex disproportionality in hospitalizations precludes a distinctive risk association in the female COVID-19 patients (27). None of the studies included had an outcome measure of investigating sex mediated differential QTc response in COVID-19 patients and given the diverse study designs, our retrospective, observational analysis lacks statistical validation. Additionally, the results could also be skewed by co-consumption of other medications and underlying co-morbidities. While validation from optimally designed trials is still required, adoption of study designs that include observation of sex mediated differential triggering of cardiac electrical activity by these drugs is warranted.

## Author Contributions

SG: research and writing. LJ: data from NYC COVID-19 trial. MB: data form Netherlands COVID-19 trial. MC: data from France COVID 19 trial. GB: women health contribution. JK: cardiology contribution. KM: project conception, coordination, and writing. All authors contributed to the article and approved the submitted version.

## Conflict of Interest

The authors declare that the research was conducted in the absence of any commercial or financial relationships that could be construed as a potential conflict of interest.

## Abbreviations

CQ: Chloroquine
HCQ: Hydroxychloroquine
AZ: Azithromycin
Os: Oseltamivir
ms: milliseconds
ΔQTc: Change in corrected QT interval
VT: Polymorphic ventricular tachycardia
TdP: torsades de pointes
Rx: Drug
Red.: Reduction in dose
Term.: Termination.
